# Recent Advances in Endosonography—Elastography: Literature Review

**DOI:** 10.3390/jcm10163739

**Published:** 2021-08-23

**Authors:** Akira Yamamiya, Atsushi Irisawa, Koki Hoshi, Akane Yamabe, Naoya Izawa, Kazunori Nagashima, Takahito Minaguchi, Masamichi Yamaura, Yoshitsugu Yoshida, Ken Kashima, Yasuhito Kunogi, Fumi Sakuma, Keiichi Tominaga, Makoto Iijima, Kenichi Goda

**Affiliations:** Department of Gastroenterology, Dokkyo Medical University School of Medicine, 880 Kitakobayashi Mibu, Tochigi 321-0293, Japan; akira-y@dokkyomed.ac.jp (A.Y.); hoshi@dokkyomed.ac.jp (K.H.); yamabe@dokkyomed.ac.jp (A.Y.); izawanao@dokkyomed.ac.jp (N.I.); n-kazu@dokkyomed.ac.jp (K.N.); takahito@dokkyomed.ac.jp (T.M.); m-yamaura@dokkyomed.ac.jp (M.Y.); y-yoshida209@dokkyomed.ac.jp (Y.Y.); ken-k@dokkyomed.ac.jp (K.K.); ykunogi@dokkyomed.ac.jp (Y.K.); sakuma-f@dokkyomed.ac.jp (F.S.); tominaga@dokkyomed.ac.jp (K.T.); mkiijima@dokkyomed.ac.jp (M.I.); goda@dokkyomed.ac.jp (K.G.)

**Keywords:** autoimmune pancreatitis, chronic pancreatitis, endoultrasonography, gastro-intestinal lesion, lymph node, pancreatic solid lesion, shear wave elastography, strain elastography, subepithelial lesion

## Abstract

Ultrasonographic elastography is a modality used to visualize the elastic properties of tissues. Technological advances in ultrasound equipment have supported the evaluation of elastography (EG) in endosonography (EUS). Currently, the usefulness of not only EUS-strain elastography (EUS-SE) but also EUS-shear wave elastography (EUS-SWE) has been reported. We reviewed the literature on the usefulness of EUS-EG for various diseases such as chronic pancreatitis, pancreatic solid lesion, autoimmune pancreatitis, lymph node, and gastrointestinal and subepithelial lesions. The importance of this new diagnostic parameter, “tissue elasticity” in clinical practice might be applied not only to the diagnosis of liver fibrosis but also to the elucidation of the pathogeneses of various gastrointestinal diseases, including pancreatic diseases, and to the evaluation of therapeutic effects. The most important feature of EUS-EG is that it is a non-invasive modality. This is an advantage not found in EUS-guided fine needle aspiration (EUS-FNA), which has made remarkable progress in the field of diagnostics in recent years. Further development of artificial intelligence (AI) is expected to improve the diagnostic performance of EUS-EG. Future research on EUS-EG is anticipated.

## 1. Introduction

Ultrasonographic elastography, based on covering strain and shear waves, is a modality used to visualize the elastic properties of tissues [1]. Reports in recent years have underscored the usefulness of extracorporeal ultrasonography (US) for ultrasound image enhancement for organs such as mammary, thyroid, and prostate glands. The usefulness of tissue elasticity in the gastrointestinal field for non-invasive and simple liver fibrosis diagnosis is widely acknowledged [2,3,4,5,6]. Technological advances in ultrasound equipment have made it possible to evaluate elastography in endosonography (EUS) [7,8]. In 2003, EUS–strain elastography (EUS-SE) was introduced. It is reportedly useful for clinical practice, for differentiating tumors, and for diagnosing chronic pancreatitis (CP). Strain elastography (SE) was initially a qualitative examination based on color patterns, but quantitative tissue elasticity diagnosis (strain ratio (SR), histogram analysis (SH)) can be performed by image processing. Nevertheless, because tissue elasticity cannot be measured using absolute values, it can only be used as a subjective measurement [9]. Shear wave elastography (SWE), which has been available since 2019, can objectively measure tissue elasticity using absolute values, in EUS. Reports of the usefulness of EUS–shear wave elastography (EUS-SWE) are emerging [10]. This review presents a summary of recent advances in EUS elastography (EUS-EG) for various diseases: CP, autoimmune pancreatitis (AIP), pancreatic solid lesions (PSL), lymphadenopathy, and gastrointestinal subepithelial lesions (GI-SEL).

## 2. Evaluating Elastography (EG) in Endoscopic Ultrasonography

### 2.1. Outline of the Evaluation Methods Using Elastography

Different evaluation methods are used for elastography of different types. Different types of EG are SE including acoustic radiation force impulse (ARFI) and SWE [9,11]. As explained herein, SE measures “strain,” which is correlated negatively with tissue elastic properties, whereas SWE measures shear wave velocity, which is correlated positively with true elastic properties. Today, both US and EUS can be used for SE and SWE [12].

### 2.2. Strain Elastography

**Figure 1:**
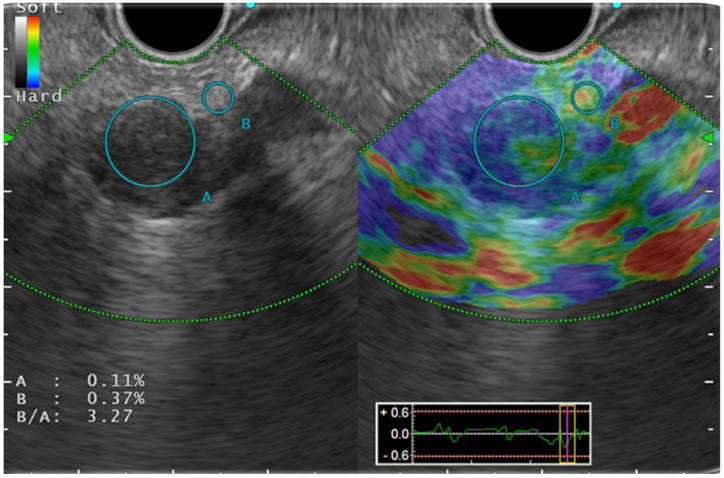
Strain elastography.

Color pattern diagnosis is the qualitative evaluation method used for SE (Figure 1). Generally speaking, color pattern diagnosis is used for major color tones of tumors (blue, hard; red, soft), and for heterogeneous or homogeneous color tones. Giovannini et al. first reported the elastic score: a color pattern diagnosis. The elastic score, color pattern, and heterogeneity of distribution of the elastography were classified into five types [13]. The quantitative evaluation methods are classified into SR and SH. The former, SR, is the ratio of the target lesion strain to the peripheral tissue strain [14]. The latter, SH, evaluates a grayscale histogram created by converting an elastography image into a gray scale of 256 tones, thereby yielding feature values [15]. The mean value, which is one of the feature values, is reportedly correlated with the degree of pathological pancreatic fibrosis. The SH results, used along with neural network analysis (NN), are particularly valuable for differential diagnosis of pancreatic cancer from pancreatic inflammatory masses [16].

### 2.3. Shear-Wave Elastography

For SWE, only the quantitative evaluation method is used. Its values are measured as the shear-wave velocity (*V*_s_) and are displayed in meters per second (m/s). After Ohno et al. conducted a clinical study to validate the suitability and usefulness of EUS-SWE, they reported the success rate of pancreatic parenchymal measurement as higher than 96%. The median accuracy of measurement was 74% [17]. For site measurement of pancreatic parenchyma hardness, the error might be caused by compression by the endoscope.

## 3. Endoultrasonography (EUS)-Elastography for Various Diseases

### 3.1. Chronic Pancreatitis/Autoimmune Pancreatitis

For CP/AIP evaluation, understanding an EUS-EG image of a normal pancreas is fundamentally important. A normal pancreas is often portrayed homogeneously in green on EUS-EG. Reportedly, the pancreas hardness increases with age [18,19].

#### 3.1.1. Chronic Pancreatitis

**Figure 2:**
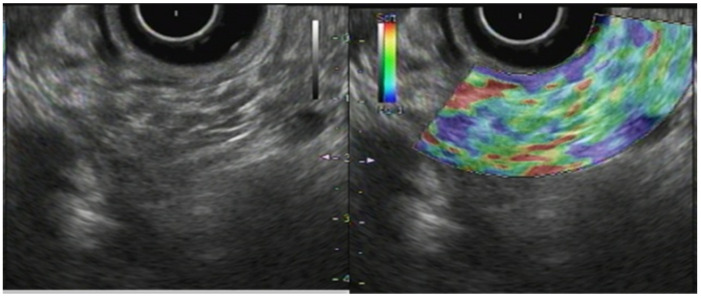
Endosonography (EUS) elastography in chronic pancreatitis.

In fact, CP has a higher degree of hardness than a normal pancreas (Figure 2). Generally speaking, CP appear as blue and heterogeneous on EUS-EG as the disease progresses, which correlates with the Rosemont classification [11,15,20]. Kim et al. have described a mean SR of 3.78 ± 1.35 for a normal pancreas and 8.21 ± 5.16 for CP. Using a cut-off value of 5.62, the sensitivity, specificity, and accuracy of SR for detecting CP were, respectively, 72%, 75%, and 75% [21]. Several published reports have described the usefulness of EUS-EG for CP (Table 1).

Yamashita et al. assessed the utility of EUS-SWE for CP diagnosis [22]. Results of that study indicate that vs. has a significant and positive correlation with the Rosemont classification and several EUS features of CP. In fact, the EUS-SWE results were consistent with CP (*V*_s_ 2.98 m/s) and were suggestive of CP (*V*_s_ 2.95 m/s). Moreover, the results were significantly higher than those found for normal tissue (*V*_s_ 1.52 m/s). Actually, EUS-SWE also showed high accuracy for diagnosing CP, with an area under the receiver operating characteristic (AUROC) curve of 0.97. The vs. cut-off of 2.19 m/s showed 100% sensitivity and 94% specificity when diagnosing CP.

However, the diagnosis of CP using the Rosemont classification is based solely on EUS findings. Moreover, its evaluation is hindered by interobserver reliability [23,24]. Earlier reports have explored the putative relation between pancreatic function tests and EUS findings. Yamashita et al., using the Japanese version of the Diagnostic Criteria for Chronic Pancreatitis (DCCP2009) for support, conducted a prospective study elucidating the relation between CP diagnosis and EUS-SWE [25,26,27,28].

The EUS-SWE results found for groups of patients with probable CP (*V*_s_ 2.78 m/s) and definite CP (*V*_s_ 3.08 m/s) were determined using DCCP2009. Consequently, the results were significantly higher than in the normal group (*V*_s_ 1.36 m/s). This method showed high accuracy for the diagnosis of CP (AUROC = 0.92), moderate accuracy for the diagnosis of exocrine dysfunction (AUROC = 0.78), and low accuracy (AUROC = 0.63) for the diagnosis of endocrine dysfunction. The vs. cut-off values of 1.96, 1.96, and 2.34 m/s for diagnosing CP, exocrine dysfunction, and endocrine dysfunction had 83%, 90%, and 75% sensitivity, respectively, and had 100%, 65%, and 64% specificity, respectively. Therefore, it was possible to demonstrate the usefulness of EUS for diagnosing pancreatic exocrine insufficiency objectively, as described in existing reports, even using EUS-SWE.

Based on those findings, EUS-EG has played an important role in CP diagnosis and pancreatic function evaluation. Moreover, the absolute values of measurements taken using EUS-SWE can enable follow-up of temporal changes of CP from an early stage [29].

#### 3.1.2. Autoimmune Pancreatitis

Ohno et al. described EUS-SWE effectiveness for assessing autoimmune pancreatitis (AIP) [12]. They found the median vs. to be significantly higher in patients with AIP (2.57 m/s) than in normal controls (1.89 m/s). Furthermore, mean vs. was found to be significantly lower in the former group after steroid treatment. These were measured as more sensitive indicators than changes in pancreatic parenchyma size or serum lgG4 levels after two weeks of treatment. These results provide new insights into pancreatic hardness, where it is high during active inflammation in AIP and decreased upon steroid therapy. Moreover, Dietrich et al. reported that pancreatic hardness might remain high after steroid treatment in AIP with a high risk of relapse. That hardness was implicated as an indicator for relapse assessment [30]. Furthermore, after they evaluated the utility of elastography for diagnosing AIP, they reported characteristic elastography patterns at the site of AIP masses and in the surrounding pancreatic tissue. Taken together, these reports support the use of EUS-EG for evaluating inflammatory activity in pancreatic inflammatory diseases such as AIP, although the study of more cases is necessary.

### 3.2. Pancreatic Solid Lesion

**Figure 3:**
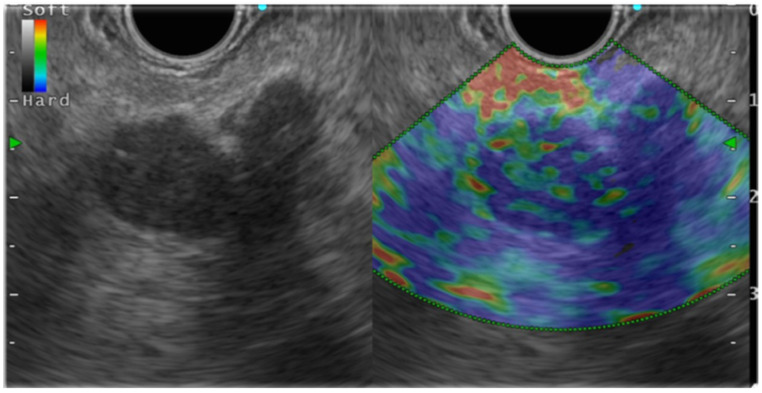
Endosonography(EUS) elastography in pancreatic ductal carcinoma.

In general, EUS-EG shows pancreatic ductal carcinoma as a heterogeneous blue color because it is harder than the surrounding pancreatic parenchyma (Figure 3). Pancreatic endocrine tumors (P-NET), portrayed as blue, are homogeneous and harder than the surrounding pancreatic parenchyma. Mass-forming pancreatitis, having lower hardness than the surrounding area and heterogeneity, is shown as green. However, as chronic pancreatitis-like changes become more intense, the lesions become heterogeneous with higher hardness (blue color) than the surrounding areas, which might make it difficult to distinguish such lesions from pancreatic cancer. Numerous reports have described studies demonstrating the usefulness of EUS-EG for PSL (Table 2, Table 3 and Table 4).

The pattern classification for PSL was first reported by Giovannini et al. in 2006 [13]. After they performed EUS-EG on 24 PSL lesions, they inferred the lesions as malignant if they were almost entirely blue, and otherwise regarded them as benign. The sensitivity and specificity for malignancy were, respectively, 100% and 67%. Subsequently, the signals were classified into five score levels according to the degrees of distortion and signal distribution. This classification was later simplified. The differential diagnostic capabilities for benign and malignant lesions were evaluated in a multicenter study. The sensitivity and specificity of B-mode alone for PSL diagnosis were 92% and 69%, respectively, whereas those of EUS-EG were 92% and 80%, respectively [31]. Since then, Janssen and Iglesias-Garcia et al. have reported on the classification based on signal heterogeneity. They reported the ability to differentiate benign and malignant pancreatic masses with high accuracy of 100% sensitivity and 85% specificity [32,33]. After Itokawa et al. used the same method, they found sensitivity of 96% and a positive diagnosis rate of 92% [14]. In addition, pancreatic diseases were classified into four groups: 1. normal control, 2. CP, 3. P-NET, and 4. pancreatic cancer. Results obtained from comparison of their scores showed that all normal control patients corresponded to Score 1. Almost all patients with pancreatic cancer corresponded to Score 5 (98%). Săftoiu et al. reported that, when the mean cutoff was set to >175 using NN, the differential diagnosis ability between pancreatic cancer and inflammatory pancreatic masses was 91–93% in sensitivity and 66–78% in specificity [16]. That finding demonstrates that EUS-EG is useful for benign and malignant diagnosis of PSL. A meta-analysis revealed the following: integrated sensitivity was 95%, integrated specificity was 67%, and the odds ratio was 42.3 (95% CI 26.9–66.46) [13,16,31,33,34,35,36,37,38,39,40,41,42]. A meta-analysis of quantitative and qualitative methods found the sensitivity and specificity for pancreatic malignancies as 98% and 63%, respectively, for qualitative evaluation, and as 95% and 61%, respectively, for quantitative evaluation, indicating that each method has high sensitivity [13,14,19,31,32,33,34,35,36,37,38,39,40,41,42,43,44,45,46,47].

Some reports have argued that CH-EUS and EUS-EG, used in combination, can improve diagnostic performance. Chantarojanasiri et al. explained the diagnostic applicability of contrast-enhanced EUS (CH-EUS) and EUS-EG for EUS diagnosis of PSL [45]. In fact, the positive diagnosis rates of pancreatic cancer were found to be 68% for CH-EUS, 65% for EUS-EG, and 76% for CH-EUS+EUS-EG. A meta-analysis of 17 reports of the literature indicated the pooled sensitivity and specificity for qualitative methods as 97% (95% CI, 0.95–0.99) and 67% (95% CI, 0.59–0.74), respectively. Other findings were also noteworthy: the pooled sensitivity and specificity for SH were 97% (95% CI, 0.95–0.98) and 67% (95% CI, 0.61–0.73), respectively; the pooled sensitivity and specificity for SR were 98% (95% CI, 0.96–0.99) and 62% (95% CI, 0.56–0.68), respectively; and the pooled sensitivity and specificity for CE-EUS were 90% (95% CI, 0.83–0.95) and 76% (95% CI, 0.67–0.84), respectively [13,14,18,19,31,33,35,37,38,39,40,41,43,44,48,49,50,51,52].

In addition, results of a multicenter study on EUS-EG for small PSLs [53] have indicated that, based on qualitative evaluation of EUS-EG images, 218 PSLs of 15 mm or less were divisible into two groups: those that were harder than the surrounding area (hard lesions) and those that were equal to or softer than the surrounding area (soft lesions). Based on data from those two groups, the sensitivity, specificity, positive predictive value, and negative predictive value for pancreatic ductal carcinoma in hard lesions were found to be 96% (95% CI, 0.87–1.00), 64% (95% CI, 0.56–0.71), 45% (95% CI, 0.40–0.50), and 98% (95% CI, 0.93–1.00), respectively. In that report, the author specifically examined the extremely high negative predictive value of 98%, inferring eventually that malignancy can be excluded with high probability when EUS-EG shows soft lesions.

Finally, the usefulness of EUS-SWE was reported specifically in 2020. Ohno et al. reported a retrospective study comparing the respective diagnostic performances of EUS-SWE and EUS-SE for 64 PSLs [54]. The vs. (m/s) values of PSLs were reported as 2.56 for mass-forming pancreatitis, 2.19 for pancreatic cancer, 1.58 for metastatic tumors, and 1.31 for pancreatic neuroendocrine neoplasms. Actually, vs. showed no significant difference based on the disease. The mean strain values were 74.5 for mass-forming pancreatitis, 47.3 for pancreatic neuroendocrine neoplasms, and 45.5 for pancreatic cancer. Based on a comparison between pancreatic cancer and mass-forming pancreatitis in terms of tissue elasticity, vs. was found to have no significant difference (*p =* 0.5687). However, the mean strain value in pancreatic cancer cases was significantly lower: 45.4 vs. 74.5: *p* = 0.0007. Unexpectedly, vs. determined from EUS-SWE was found to have no significance among PSLs of different types.

The method of evaluating EUS-EG including EUS-SWE and differentiation of PSL was reviewed as follows: the evaluation methods of EG and their diagnostic abilities (sensitivity and specificity) were 82% (95% CI, 0.77–0.86) and 70% (95% CI, 0.64–0.76) for color pattern diagnosis, 94% (95% CI, 0.90–0.97) and 87% (95% CI, 0.81–0.92) for SR, 92% (95% CI, 0.90–0.94) and 79% (95% CI, 0.75–0.82) for SH, and 90 (95% CI, 0.82–0.95) and 82% (95% CI, 0.57–0.72) for SWE [2,13,14,19,21,22,32,34,37,38,39,40,41,42,43,45,47,54,55,56,57,58,59,60,61,62,63]. Therefore, EG and evaluation methods of many types are expected to be available: each has its own high diagnostic ability for PSL. Histological diagnosis by EUS-fine needle aspiration (EUS-FNA) is often necessary for the diagnosis of PSL; EUS-EG is useful as an adjunct diagnosis or as a method of screening before histological diagnosis.

### 3.3. Lymphoadenopathy

In addition to helping discriminate benign diseases, differential diagnosis of the lymph node (LN) can provide information for the staging of malignant diseases. Therefore, early and correct differentiation of benign and malignant LN is crucially important for clinical decision-making. For the differential diagnosis of lymph nodes, EUS-EG shows benign nodes to be as hard (green) as their homogeneous surroundings. It also shows malignant nodes to be harder (blue) than their homogeneous surroundings.

Giovanninni et al. first reported the differentiation of benign and malignant enlarged LN based on pathological diagnosis by EUS-guided fine needle aspiration (EUS-FNA) in 2006 [13]. They described sensitivity of 100% and specificity of 50% by pattern classification using the elastic score. A multicenter study later indicated sensitivity of 91% and specificity of 83% for the same pattern classification, which was significantly superior to B-mode diagnosis [31]. By contrast, Saftoiu et al. used SH analysis to differentiate between benign and malignant LN. Based on those findings, they reported high diagnostic performance: 92% sensitivity, 94% specificity, and a 93% positive diagnosis rate [64]. From one meta-analysis, integrated sensitivity of 88% and integrated specificity of 85% were found from 368 cases described in seven reports [13,31,64,65,66,67,68,69]. Therefore, EUS-EG is a promising, non-invasive method for differential diagnosis of malignant LNs. It might be a valuable supplemental method to EUS-FNA.

### 3.4. Gastrointestinal (GI)-Subepithelial Lesions

For diagnosis of GI-SELs, distinguishing between gastrointestinal stromal tumor (GIST) and other gastrointestinal mesenchymal tumor such as leiomyoma and Schwannoma is crucially important. Pathological diagnosis is possible using EUS-FNA, but results of EUS-FNA for SEL of 20 mm or less are not always reliable [70,71,72]. Therefore, differentiation by imaging diagnosis is valuable for the management of GI-SELs.

Tsuji et al. used the elastic score to classify patterns of 25 gastric SEL [71]. Their findings indicate that six out of nine gastrointestinal stromal tumors (GIST) had a score of 4, and that three out of nine had a score of 5 compared with the pathological diagnosis and elastic score, indicating that GIST are depicted as “hard” tissues compared with other SEL. In contrast, Ignee et al. reported difficulty in differentiating GIST from benign leiomyoma by pattern diagnosis using an elastic score [72]. The eventual utility of EUS-EG in this field remains unclear. Nevertheless, additional studies are warranted because of its high importance.

## 4. Limitations of EUS Elastography

Major limitations of EUS-EG include intra-observer and inter-observer variation from endoscopists with varying experience. Skill and experience among these valuable professionals is crucially important to obtain reproducible results [73]. Secondly, the pancreatic cancer detection rate of EUS-EG depends on tumor size, tumor volume, localization, and histological type. Furthermore, for EUS, some problems persist with accuracy, such as the need to rely on the heartbeat to compress the ultrasound probe [74].

## 5. Future Prospects of EUS-EG

As described in this review, non-invasive EUS-EG imaging method produces a variety of information. Nevertheless, endosonographers cannot be blinded to B-mode images obtained using EUS. Therefore, the possibility exists of bias in the accuracy of EUS-EG [75,76]. In addition, most studies address only lesions confirmed as malignant or benign. They exclude indeterminate lesions, which might unfairly increase the sensitivity and specificity of this technique. Currently, EUS-FNA/FNB is used widely as a fundamentally important examination method for the definitive diagnosis of not only PSL but also of lymphadenopathy [77,78]. However, a certain number of false-negative and false-positive cases persist. Their improvement is required. EUS-EG directs the appropriate puncture site, which might increase the accuracy of EUS-FNA/FNB. It is noteworthy that EUS-EG is especially useful in facilities where rapid onsite evaluation is not possible. In addition, the possibility of needle tract seeding should always be considered with EUS-FNA/FNB [79,80]. From this point of view, EUS-EG is important because it can be performed non-invasively. It is also expected that EUS-SWE will be compatible with all processors. The application of artificial intelligence (AI) to AI diagnosis and EUS-EG might lead to further improvement in diagnostic performance. Future research on EUS-EG is anticipated.

## 6. Conclusions

The development of EUS imaging has followed the progress in technologies applied for ultrasound equipment. The importance of this new diagnostic parameter, “tissue elasticity” in clinical practice might be applied not only to the diagnosis of liver fibrosis but also to the elucidation of the pathogeneses of various gastrointestinal diseases, including pancreatic diseases, and to the evaluation of therapeutic effects. We earnestly hope that EUS-EG can be exploited further by virtue of software and endoscopic hardware to become an even stabler and simpler diagnostic method.

## Figures and Tables

**Table 1:** Usefulness of endosonography–elastography (EUS-EG) in chronic pancreatitis.

	Year	Number	Evaluation Method	
Kuwahara [11]	2017	96	Histogram	Indeterminate for CP: 73.2 Suggestive of CP: 63.7 Consistent with CP: 56.1
Kim [21]	2017	67	Strain ratio	Cut-off value: 5.62
Yamashita [22]	2020	52	Shear wave	Cut-off value: 2.19 m/s
Yamashita [27]	2021	40	Shear wave	Cut-off value: 1.96 m/s

CP, chronic pancreatitis.

**Table 2:** Usefulness of color pattern in pancreatic solid lesion.

	Year	Number	Prevalence of PDAC (%)
Giovannini [16]	2006	24	58
Jassen [32]	2007	33	73
Hirche [57]	2008	70	66
Giovannini [31]	2009	121	60
Iglesias-Garcia [33]	2009	130	59
Itokawa [14]	2011	109	66
Lee [58]	2013	35	43
Hocke [41]	2012	58	33
Chantarojanasiri [45]	2017	136	69
Ignee [53]	2018	218	51

PDAC, pancreatic ductal adenocarcinoma.

**Table 3:** Usefulness of the strain ratio in pancreatic solid lesion.

	Year	Number	Prevalence of PDAC (%)	Cut-Off Value
Dawwas [39]	2012	111	71	6.04
Figueriedo [40]	2012	47	70	8
Havre [42]	2014	48	23	3.05
Kongkam [35]	2015	38	61	3.17
Mayerle [44]	2016	91	53	10
Rusebtemovic [59]	2017	149	36	7.59
Okasha [47]	2017	172	56	7.8
Kim [21]	2017	157	57	8.86

PDAC, pancreatic ductal adenocarcinoma.

**Table 4:** Usefulness of histogram in pancreatic solid lesion.

	Year	Number	Prevalence of PDAC (%)	Cut-Off Value
Saftoiu [37]	2010	54	61	175
Iglesias-Garcia [34]	2010	62	79	0.05 (elastic value)
Saftoiu [38]	2011	258	82	170
Opacic [43]	2015	105	50	86

PDAC, pancreatic ductal adenocarcinoma.

## Data Availability

No new data were created or analyzed in this study. Data sharing is not applicable to this article.
